# Proteomic Approaches to Defining Remission and the Risk of Relapse in Rheumatoid Arthritis

**DOI:** 10.3389/fimmu.2021.729681

**Published:** 2021-11-18

**Authors:** Liam J. O’Neil, Pingzhao Hu, Qian Liu, Md. Mohaiminul Islam, Victor Spicer, Juergen Rech, Axel Hueber, Vidyanand Anaparti, Irene Smolik, Hani S. El-Gabalawy, Georg Schett, John A. Wilkins

**Affiliations:** ^1^ Section of Rheumatology, Department of Internal Medicine, University of Manitoba, Winnipeg, MB, Canada; ^2^ Manitoba Centre for Proteomics and Systems Biology, University of Manitoba and Health Sciences Centre, Winnipeg, MB, Canada; ^3^ Department of Biochemistry and Medical Genetics, University of Manitoba, Winnipeg, MB, Canada; ^4^ Department of Computer Science, University of Manitoba, Winnipeg, MB, Canada; ^5^ Department of Medicine, Friedrich-Alexander University Erlangen-Nuernberg and Universitaetsklinikum Erlangen, Erlangen, Germany

**Keywords:** rheumatoid arthritis, disease activity, outcomes research, treatment, proteomics

## Abstract

**Objectives:**

Patients with Rheumatoid Arthritis (RA) are increasingly achieving stable disease remission, yet the mechanisms that govern ongoing clinical disease and subsequent risk of future flare are not well understood. We sought to identify serum proteomic alterations that dictate clinically important features of stable RA, and couple broad-based proteomics with machine learning to predict future flare.

**Methods:**

We studied baseline serum samples from a cohort of stable RA patients (RETRO, n = 130) in clinical remission (DAS28<2.6) and quantified 1307 serum proteins using the SOMAscan platform. Unsupervised hierarchical clustering and supervised classification were applied to identify proteomic-driven clusters and model biomarkers that were associated with future disease flare after 12 months of follow-up and RA medication withdrawal. Network analysis was used to define pathways that were enriched in proteomic datasets.

**Results:**

We defined 4 proteomic clusters, with one cluster (Cluster 4) displaying a lower mean DAS28 score (p = 0.03), with DAS28 associating with humoral immune responses and complement activation. Clustering did not clearly predict future risk of flare, however an XGboost machine learning algorithm classified patients who relapsed with an AUC (area under the receiver operating characteristic curve) of 0.80 using only baseline serum proteomics.

**Conclusions:**

The serum proteome provides a rich dataset to understand stable RA and its clinical heterogeneity. Combining proteomics and machine learning may enable prediction of future RA disease flare in patients with RA who aim to withdrawal therapy.

## Highlights

- Serum proteomics defines clinically relevant clusters within a cohort of stable RA patients
- Machine learning and proteomics may identify individuals at highest risk for future disease flare
- Despite meeting criteria for remission, clinically detectable disease is associated with a serum proteomic signature in stable RA

## Introduction

Rheumatoid Arthritis (RA) is a systemic autoimmune disease that is characterized by inflammation of synovial joints (1). Modern RA therapy is initiated early and escalated aggressively using a treat-to-target approach to try an obtain disease remission (2). The development of both targeted treatments and combination regimens continues to improve expected outcomes for patients. Encouragingly, clinical remission, defined by multiple measures of disease activity (3), has become a realistic expectation for most patients with RA. Recent registry data of RA cohorts consistently show that DAS28 (Disease Activity Score) remission is achieved in about 50% of patients (4), a number that may be increasing over time (5).

Patients with RA who are able to achieve disease remission using standard therapy are not well studied, given their lack of disease activity and need for treatment changes. The main issue facing these patients is whether or not to remain on their treatment, or risk withdrawal and the potential for disease flare. There are many prospective studies that have demonstrated successful Disease Modifying Anti-Rheumatic Drugs (DMARD) withdrawal in patients in clinical remission (6–9) but the determinants of maintaining remission status after medication withdrawal are poorly defined (10). Unfortunately, given the limited understanding of the pathological mechanisms that drive subclinical disease, clinicians are left to guess which of their patients might sustain remission using less aggressive therapy.

Technological advances in high-throughput proteomics have allowed for an improved understanding of disease processes and biomarker discovery (11). Although mass spectrometry tends to dominate this evolving field, broad-based targeted proteomics has its own advantages, including simplified sample preparation and user-friendly output data (12). Our group has previously defined protein sets that are associated with future disease flare from pre-clinical RA by coupling machine learning with proteomic approaches (13). Indeed, leveraging omics approaches to resolve heterogeneity in common diseases remains a distinct challenge in clinical medicine (14), though this has not been systematically undertaken in a stable RA cohort.

The RETRO (15) (Reducing therapy in rheumatoid arthritis patients in ongoing remission) study is a prospective randomized trial which enrolled patients who had achieved disease remission with conventional RA therapy. One of the aims of this study is to define disease recurrence in patients with RA when either continuing or reducing their medications. It was previously shown in this trial that positive anti-citrullinated antibody (ACPA), and other biomarkers (16, 17) are associated with an increased likelihood of disease relapse. In spite of these studies, there is little understanding of the underlying biological mechanisms that are active in stable RA. If differences within RA patients in remission can be more clearly defined, there may be an enhanced understanding of the spectrum of RA pathogenesis, along with improved personalized clinical approaches surrounding the withdrawal of therapy. We hypothesized that high-throughput proteomics (18) would help identify underlying biological heterogeneity that might provide insights into mechanisms underpinning future disease flare. Our aim was to explore how the serum proteome shapes the underlying clinical experiences of stable RA patients.

## Methods

### Patients and Inclusion Criteria

RETRO is a multicentre, randomized, open, prospective, controlled parallel-group study. Details of the study are described in the original publication (15). The objective of the study is to evaluate tapering or discontinuation of DMARDs in patients with RA. All enrolled patients fulfilled the 2010 American College of Rheumatology (ACR) criteria for RA (19). Patients had to have sustained clinical remission defined by the Disease Activity score (DAS28 < 2.6) criteria for at least 6 months (20). Ethics committee of the Friedrich-Alexander-University of Erlangen-Nuremberg approval was granted.

### Treatment and Follow-up

Patients were randomized to one of three arms: Arm 1 continued with existing DMARD regimen at full dose for 12 months, arm 2 reduced the dose of all DMARDs by 50%, while arm 3 reduced the dose of all DMARDs by 50% in the first 6 months, then discontinued all medications. Relapse of disease was defined as a DAS28-ESR score greater than 2.6. Participants were assessed for clinical disease activity every 3 months until month 12.

### Assessment of Demographic and Disease-Specific Parameters

Age and sex were recorded in all patients. Disease duration, tender joint count (68), swollen joint count (66), patient visual analogue scale (VAS) for pain and patient global were assessed and recorded. C-reactive protein (CRP), ESR, Rheumatoid Factor (RF), ACPA, DAS28-ESR and Health Assessment Questionnaire (HAQ-DI) were recorded.

### SOMAscan

SOMAscan is a proteomics assay that measures 1307 proteins using an aptamer library. This high-throughput proteomics assay has been used in recent publications to study the aging proteome (21, 22) along with other human diseases (23). 130 baseline serum samples were available from the RETRO study. Briefly, a library of aptamers were incubated with serum, and those that bind are isolated and hybridized to DNA microarray for detection. The identity and relative concentration of the detected proteins are revealed by localization and fluorescence intensity. Protein quantification is reported as relative fluorescence units (RFU), an arbitrary value. In general, agreement between aptamer and antibody-based assays is high (24). Further details regarding the SOMAscan assay are available (18).

### Statistical Analysis

Descriptive results (**Table 1**) are stated in means and standard deviation. SOMAmer protein expression RFU values for the study patients were transformed into a log2 scale for differential analysis (**Supplemental File 2**). Batch effect was removed in our SOMAmer data using internal controls within each plate to adjust proteomic intensity as per standard SOMAscan protocols. Batch effect was assessed between plates and determined to require no further correction. Data was loaded and analyzed in the R (v3.5.3) environment unless otherwise stated. Missing clinical data was imputed using multiple imputation by chained equations (MICE) (25). Differential analysis between groups was undertaken using linear modeling with the package *LIMMA (* 26). GO pathways analysis was performed using clusterprofiler (27). Graphs were generated using the *ggplot2* package. Correlation analyses were performed for select proteins using Pearson correlation. Multi-dimensional scaling (MDS) was used for dimension reduction on all SOMAscan proteins.

**Table 1:** Baseline characteristics of the patients, split by proteomic cluster.

Characteristics	Total (n = 130)	Cluster 1 (n = 34)	Cluster 2 (n = 12)	Cluster 3 (n = 46)	Cluster 4 (n = 38)
Age	55.2 (13.1)	52.7 (14.6)	54.1 (13.7)	54.7 (11.7)	58.6 (13.1)
Females, %	56.2%	67.6%	66.7%	56.5%	42.1%
Disease Duration (years)	6.8 (7.0)	7.9 (6.5)	8.6 (9.3)	6.9 (6.3)	4.9 (7.3)
DAS-28 (ESR)	1.7 (0.68)	1.93 (0.60)	1.73 (0.74)	1.71 (0.65)	1.51 (0.71)
ACR/EULAR remission, %	76.6%	67.7%	66.6%	88.9%	72.9%
HAQ, units	0.12 (0.32)	0.11 (0.17)	0.08 (0.12)	0.16 (0.46)	0.09 (0.26)
Positive RF, %	56.2%	73.5%	50.0%	52.2%	47.3%
Positive ACPA, %	57.7%	67.6%	66.7%	55.6%	50.0%
*Biological DMARD use, % (N)	40.0%	38.2%	25.0%	47.8%	36.8%
Flare, %	37.7%	38.2%	16.7%	43.5%	36.8%

ACPA, anticitrullinated protein antibody; ACR, American College of Rheumatology; CRP, C-Reactive protein; DAS-28, disease activity score-28 (based on ESR); DMARDs, disease modifying antirheumatic drugs; ESR, erythrocyte sedimentation rate; EULAR, European League Against Rheumatism; HAQ, Health Assessment Questionnaire; RF, Rheumatoid Factor; VAS, Visual analogue scale.

*Tumor necrosis factor inhibitors and tocilizumab.

The 200 most variable proteins measured by coefficient of variation were used to determine optimal number of clusters ranging from k = 2 to 10 and identify sample clusters using the R package *Consensusclusterplus*. We used 80% protein resampling and 80% patient resampling and selected Pearson as our distance function. Multinomial logistic regression implemented in R package *glmnet* was used to identify clinical variables that are independently associated with cluster assignment. Sliding window analysis of DAS28 scores and protein expression was performed using a previously published algorithm, DE-SWAN (28). Briefly, this algorithm analyzed serum protein expression across quintiles of DAS28 scores using linear modeling, while adjusting for baseline demographic factors, in this case age and sex. A protein expression score was developed on proteins that correlated with DAS28 which were identified by DE-SWAN. We filtered the proteomic data on the 34 score members, scaled the data by the mean and standard deviation, and multiplied by 1 (positively associated with DAS28) or -1 (negatively associated with DAS28) for each protein. The final score was the mean expression of all 34 proteins for each patient. We randomly generated 5000 data sets with 34 randomly selected proteins in each set to evaluate the significance of the association score.

### Machine Learning Classification Algorithm

We applied two supervised machine learning (ML) techniques to develop algorithms to classify flare or remission based on serum proteomics. The first approach we used is XGBoost (Extreme Gradient Boosting), which employs a regularization term to overcome the overfitting (29). The second approach is the LASSO model (30), which was used as a baseline to compare its performance with that of XGBoost. Data was loaded into Python, and samples were randomly split into a training (n = 104, 80% of the samples) and test (n = 26) set. The training set was used to train and tune the parameters in the two models and the test set was used evaluate the models’ performance, which were measured by the area under of the curve (AUC) of receiver operating characteristic (ROC), accuracy, sensitivity and specificity. To increase the interpretability of the XGBoost model to predict the flare status of a given sample, we used SHAP values (Shapley Additive Explanation) (31). A higher SHAP value of a given feature in the model represents its strong influence on the model output. The final model parameters we used in the XGBoost are as follows: learning_rate = 0.01, max_depth = 3, subsample = 0.6, colsample_bytree = 0.7, n_estimators = 100, gamma = 0.0, reg_alpha = 0.5, the parameters used in the LASSO model is as follow: cost=1.17 and max_iterations = 5000. We used 5-fold cross-validation to get the optimal hyperparameters.

### Study Cohort

Baseline characteristics for 130 patients enrolled in the RETRO study are found in **Table 1**. Overall, the group had maintained clinical remission for 16.6 (16.2) months and mean disease duration of over 6 years. 57.7% of the patients were ACPA positive, while 40.0% required biologics to achieve remission. 76.6% of patients had achieved the most stringent definition of remission [ACR/EULAR remission (32)]. After 12 months of follow-up, 62.3% of the overall population remained in clinical remission (50% in those undergoing withdrawal).

## Results

### Hierarchical Clustering on Serum Proteins Identifies Heterogeneity Amongst Stable RA Patients

**Figure 1:**
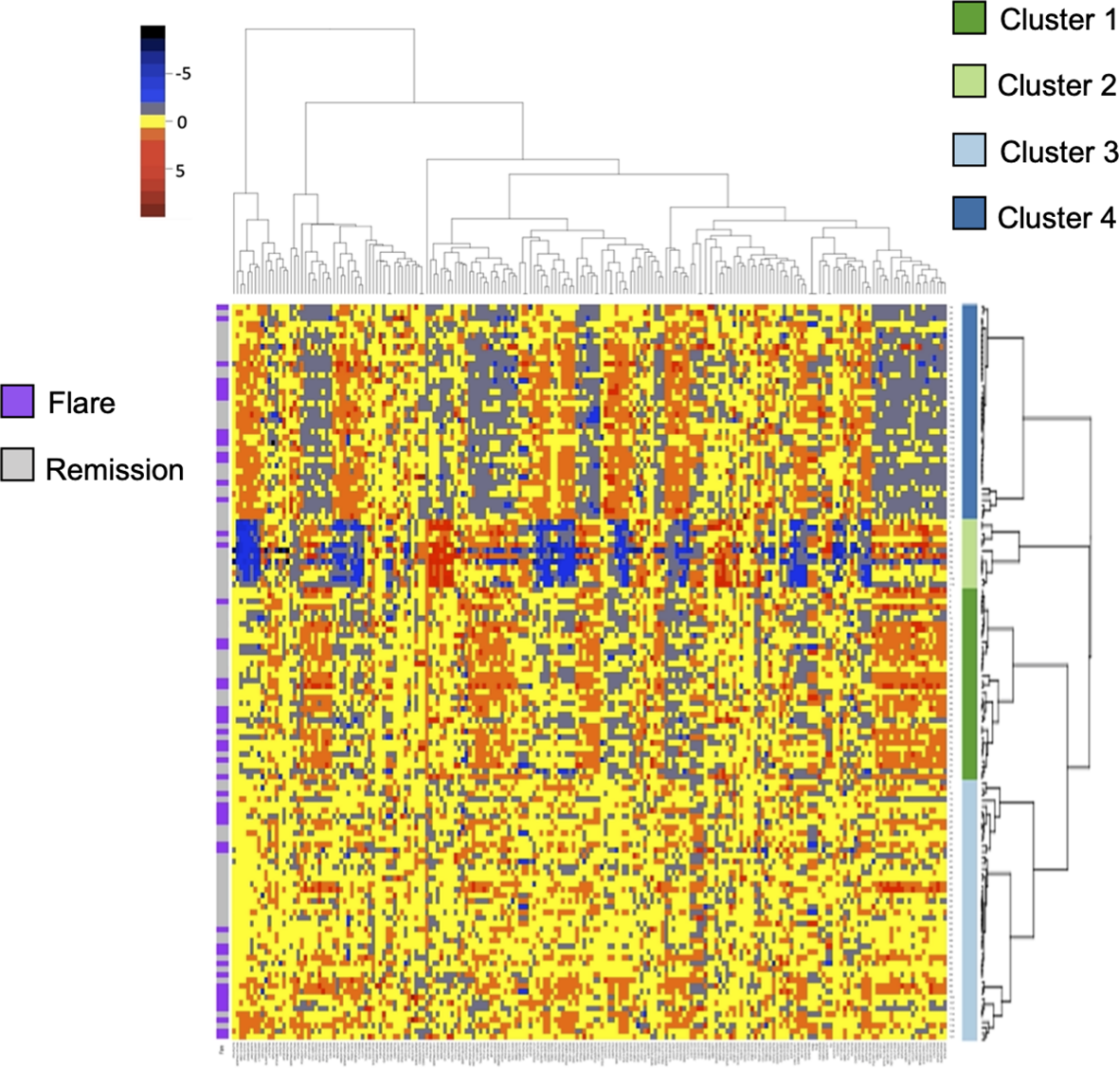
Heatmap and hierarchical clustering of 200 serum proteins in stable RA. Protein expression is scaled and colored by relative expression. Each column is a protein and each row is a patient. Clustering is shown in both dimensions. Flare or remission is annotated in purple or grey to the left of the graph.

Given the paucity of data aimed at understanding subclinical disease activity in RA patients who achieve remission, we sought to explore underlying heterogeneity using serum proteomics in this established cohort. We quantified over 1300 serum proteins from 130 RETRO patients at their baseline visit, all of whom were in stable clinical remission (DAS28 < 2.6). We hypothesized that despite the clinical similarities amongst individuals within this cohort, proteomic differences may provide important insights by identifying sub-clusters of patients. We applied consensus clustering to assign individuals to one of the 4 clusters (**Figure S1**) and clustered scaled protein expression by hierarchical clustering, which can be seen in **Figure 1**. MDS analysis revealed separation of the hierarchical clusters (**Figure S2**).

Baseline characteristics split by cluster are listed in **Table 1**. We found no differences in sex, age, biologic use, or serological status across our 4 proteomic clusters. Cluster 4 had significantly lower DAS28 scores compared to the remaining clusters. With respects to future flare, Cluster 2 trended toward lower rates relative to the remaining clusters, however this did not reach statistical significance (16.7% vs 39.8%, p = 0.21). To assess this association by multinomial regression, we assigned Cluster 2 as the reference cluster and found that that Cluster 3 had higher odds of flare (OR 5.6, 0.97 to 33.06, p = 0.05), relative to Cluster 2 with similar trends observed for Cluster 1 and Cluster 4 (**Table S1**). Indeed, no clear distinction between individuals who developed future flare was observed in the MDS plot (**Figure S2**). Overall, these results suggest that global proteomic clusters within a clinically homogenous cohort can be identified but are associated with current clinical status rather than future outcomes.

### Machine Learning Classifies Future Flare Using Baseline Serum Proteomics

**Figure 2:**
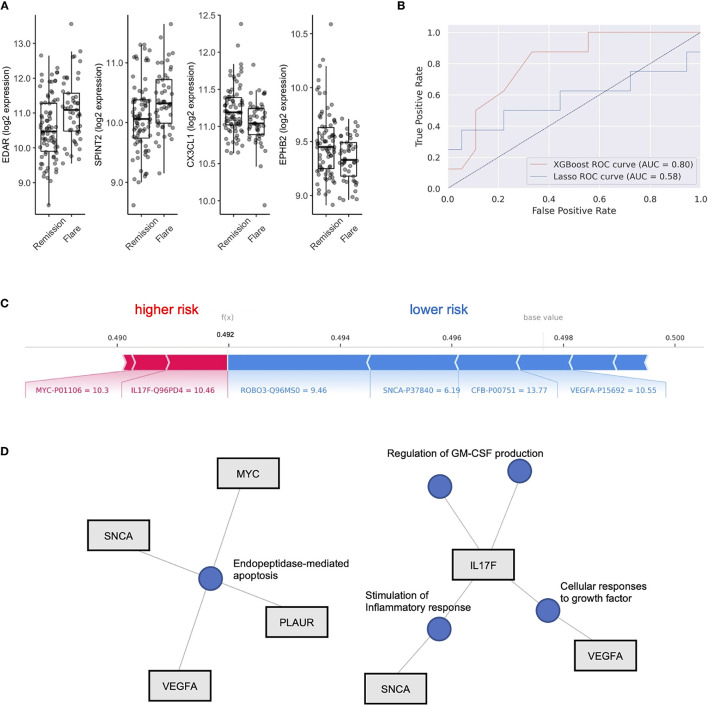
XGboost machine learning to identify flare or remission in stable RA patients. **(A)** Box plots of EDAR, SPINT2, CX3CL1 and EPHB2 split by individuals who remained in Remission or Flare. **(B)** Receiver operator curves (ROC) of 2 machine learning models, XGboost and LASSO, trained on serum proteome to classify flare or remission. AUC is representative of test set cohort parameters. **(C)** Bar plot of model features that impact risk of flare or remission in the XGboost model with log2 expression and Uniprot ID annotated for each protein member. Feature importance is represented by relative size of bar. Values for different proteins represent their original values in the dataset for that particular sample. The base value means the average of the prediction scores, and 0.5 is the cutoff threshold to select a Flare status. **(D)** Gene concept plots derived from XGboost protein features. Each node represents a GO pathway with proteins connected by edges. EDAR, Ectodysplasin A receptor; SPINT2, Serine peptidase inhibitor; CX3CL1, Fractalkine; EPHB2, Ephrin type-B receptor 2. SNCA, Synuclein alpha; PLAUR, Plasminogen Activator; VEGFA, vascular endothelial growth factor A; MYC, Myc proto-oncogene protein; IL17F, Interleukin 17F; ROBO3, Roundabout homolog 3; CFB, complement factor B.

We next aimed to use the serum proteome to identify biomarkers associated with future disease flare in stable RA, given that clustering did not clearly associate with risk of flare. We identified DEP’s between these groups (**Table S2**) and observed upregulation in Ectodysplasin A receptor (EDAR, FC = 1.20) and Serine peptidase inhibitor (SPINT2, FC 1.1), and downregulation of Fractalkine (CX3CL1, FC 0.95) and Ephrin type-B receptor 2 (EPHB2, FC 0.95) in individuals who eventually went on to flare (**Figure 2A**). However, after adjustment for multiple comparisons, none of the differentially expressed proteins reached statistical significance. This suggests that although subtle differences exist in the serum proteome between individuals who experience future flare, it’s unlikely that singular biomarkers accurately predict this outcome in this population.

To test this hypothesis, we explored the use of two machine learning algorithms, LASSO and XGBoost, to build predictive models that classify future flare using baseline serum proteomics. We generated two models, both of which were validated on a test cohort (20% of total cohort). The LASSO model achieved 69.2% accuracy, with an AUC of 0.58, along with sensitivity of 0.5 and specificity of 0.78 based on the test cohort (Features in **Table S3**). XGboost delivered a model with higher specificity (0.78) than sensitivity (0.63) and an overall accuracy of 73.1% with an AUC 0.80. Therefore, we found that the XGboost model outperformed the LASSO model by the metric area under the curve (**Figure 2B**, AUC, 0.80 *vs* 0.58), accuracy (73.1% *vs* 69.2%) and sensitivity (0.63 *vs.* 0.5).

To interpret this XGBoost model, we explored the impact of essential features in terms of SHAP values on the classifier’s output for a single prediction which are shown in **Figure 2C**. We identified Interleukin 17F (IL17F) and Myc proto-oncogene protein (MYC) expression as indicators of future flare, while Roundabout homolog 3 (ROBO3), Synuclein alpha (SNCA), complement factor B (CFB) and vascular endothelial growth factor A (VEGF-A) expression were indicators of sustained remission (**Figure 2C** and **Figure S3**). Given the small number of proteins that derived our boosted model, we next explored whether there were any functional links between these proteins. We developed gene concept plots to identify potential protein interactions and found that SNCA, MYC, VEGFA and PLAUR were connected by a single pathway, *endopeptidase mediated apoptosis*. We then analyzed IL17F restricted networks, given its conflicting role in RA (33–36), and that its expression was associated with future flare in our model. We found that IL17F interacted with VEGFA though *growth factor* function, and with SNCA through common effects on the *inflammatory response* (**Figure 2D**). IL17F independently regulated *GM-CSF production*, a key driver of RA disease activity through the recruitment of neutrophils (37). Overall, this network analysis suggests that cellular apoptosis and GM-CSF production may be associated with future disease flares in RA patients who are otherwise stable.

### Disease Activity in Stable RA Is Reflected in the Serum Proteome

**Figure 3:**
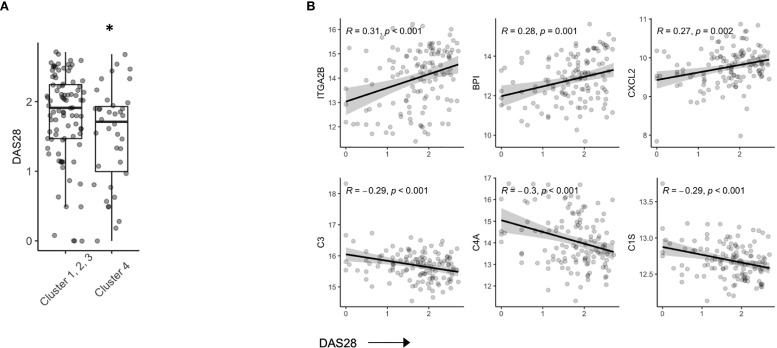
Serum proteins are associated with DAS28 in stable RA. **(A)** Box plots of DAS28 disease scores in patients, split by cluster assignment (p = 0.03). **(B)** Correlation plots of DAS28 and serum protein expression of ITGA2B, BPI, CXCL2, C3, C4A, C1S. *p < 0.05.

In our hierarchical clustering, we observed a lower mean DAS28 score in Cluster 4 compared to the remaining 3 clusters (**Figure 3A**). RA patients who achieve DAS28 defined remission often have residual disease activity, however, since this population is not typically the focus of translational studies, little is known regarding biomarkers that are reflective of ongoing disease activity. Indeed, we found that several protein members correlated with DAS28 score (**Figure S4**), including Integrin alpha 2B (ITGA2B), Bactericidal permeability-increasing protein (BPI) and chemokine ligand 2 (CXCL2, Pearson R, all p value < 0.01). Further, complement proteins (C3, C4A, C1S) were all negatively associated with DAS28 score, suggesting activation and consumption of complement proteins (**Figure 3B**) were indicators of disease. There was no indication that these parameters varied based on ACPA status (**Figure S5**).

**Figure 4:**
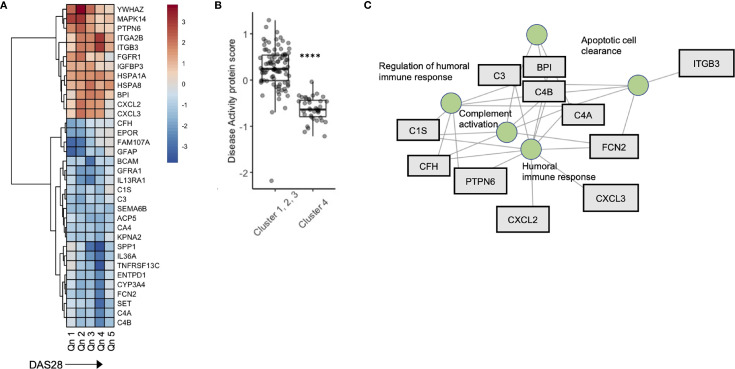
A serum proteomic signature is associated with DAS28 in stable RA. **(A)** Sliding window analysis of disease activity (Quintiles x-axis) and 34 proteins that vary with DAS score after controlling for *Age* and *Sex*. Heatmap is colored on coefficient relationship to DAS score **(B)** Box plots of disease activity protein score split by cluster assignment. **(C)** Gene concept plots derived from disease activity protein score. Each node represents a GO pathway with proteins connected by edges. ITGA2B, Integrin alpha 2B; BPI, Bactericidal permeability-increasing protein; CXCL2, Chemokine ligand 2; C3, Complement C3; C4A, Complement 4A; C1S, Complement 1S. ****p < 0.00001.

To further explore the relationship between disease activity and serum biomarkers, we developed a sliding window model (SWAN) which examined protein variability across DAS28 quintiles, after controlling for *Age* and *Sex*. Across DAS28 a total of 34 proteins varied significantly with disease activity (**Figure 4A**). We used these 34 protein members to annotate a meta-protein expression score (the mean expression profiles of the 34 proteins), which correlated with DAS28 (R = 0.45, p < 0.001, **Figure S6**). To test the robustness of this finding, we sampled 5000 random sets with 34 proteins in each set and correlated their mean expression with DAS28 scores. We found a range of -0.37 – 0.27, associated with a low probability (0) that the correlation of 0.45 would occur by chance (**Figure S7**). This protein disease activity score was significantly lower in Cluster 4 compared to the remaining 3 clusters (**Figure 4B**), concordant with their lower DAS28 scores. Gene concept plots revealed that these 34 proteins interacted through nodes that included *humoral immunity, apoptosis*, and *complement activation* (**Figure 4C**). Overall, these data suggest that stable RA disease activity is marked by complement consumption and humoral immune responses, which is reflected in a serum protein signature that is detectable in patients with stable RA.

## Discussion

With the advent of multiple targeted therapies, alone and in combination, most RA patients should reasonably expect to achieve low disease activity or clinical remission status. To date, few studies have sought to understand the heterogeneity of the biological pathways underpinning clinical remission in this rapidly expanding population of RA patients. When, and in whom to attempt withdrawal of therapy has become a compelling clinical question. On the one hand, there is justifiable concern regarding reactivation of systemic and articular inflammation. On the other hand, ongoing use of DMARDs and/or biologics is associated with increased risk of infectious complications (38), malignancy (39) and cost (40). Strategies for successful taper however remain ill-defined and, importantly, lack precision (6, 41, 42). The RETRO clinical trial has previously generated predictive models that were based on clinical parameters and serum studies (16, 17). ACPA seropositivity appears to be an important indicator for increased risk of future relapse (17), while clinical parameters have modest predictive value even when combined with advanced machine learning techniques (43). It remains unclear if these indicators are clinically applicable and generalizable to a wide range of RA patient populations. The focus of this study was to use a broad-based serum proteomic approach to better understand the underlying heterogeneity amongst RA patients who are in sustained clinical remission, prior to their participation in a clinical trial of therapy withdrawal. Our results identify proteomic signatures reflecting biological mechanisms that are associated with ongoing disease stability off therapy, or alternatively, the risk of future disease relapse.

Our XGboost model suggested that individual circulating serum biomarkers are unlikely, on their own, to be predictive of future stability or relapse after therapy withdrawal. In spite of this, combinations of proteomic biomarkers identified by the machine learning achieved relatively high AUC and accuracy in predicting outcomes. Indeed, this is a testament to the power of rapidly evolving machine learning algorithms that are being developed for many clinical problems (44). Network analysis of proteins derived from machine learning suggested that inflammatory forms of cellular death was an indicator for risk future disease flare. Indeed, apoptosis is escaped by pathogenic fibroblast-like synoviocytes and likely contributes to their aggressive and hyperplastic phenotype in RA (45) and this may point to systemic FLS as a potential source of these proteins (46). Hierarchical clustering identified proteomic clusters which were defined, in part, by clinical characteristics. Cluster 4 represented a patient group with lower DAS28 scores amongst the remaining patient cohort. Our disease activity signature, found to be lower in Cluster 4, suggested that elements of humoral immunity and complement activation might facilitate disease activity in otherwise stable RA patients. This suggests that activating pathways differentially regulate disease activity in this subset of RA patients, as many of the well-known disease activity markers in RA suggest that innate immune responses associate with DAS28 scores (47–49).

The analyses we undertook utilized the SOMAscan aptamer-based technology to interrogate 1307 distinct serum proteins. Although this provided us with a robust array of biomarkers, it is well recognized that these represent only a fraction of the human proteome, and that larger arrays that span a larger proportion of the circulating proteome may help generate even more accurate predictive algorithms. Moreover, there remains an incomplete understanding of how aptamer-based detection of each individual analyte correlates with other detection methodologies such as those that are antibody based (24). Due to our modest sample size, we observed strongly statistically significant association although R values related to DAS28 scores were all below 0.3. However, we expect the R values may increase using a larger sample size while the significant association will be still held. Finally, SOMA proteins may bias over-representation analysis based on the selected proteins which are included in the set. Notably, network analysis was used in this study to connect proteins of interest through biological nodes. These results would not be impacted by inherent bias in the SOMA protein set.

In conclusion, we applied an unsupervised, high-throughput proteomics assay to delineate biomarkers and pathways that reflect the biological heterogeneity present in RA patients who are collectively deemed to be in stable clinical remission. Based on this, we used supervised machine learning to develop robust models that predicted ongoing disease stability after therapy withdrawal as opposed to future disease flare. Although it is premature to try and define the potential clinical utility of these models, they do provide an important impetus for further studies that aim to further define a biological definition of remission in RA patients that can ultimately guide clinical decision making.

## Data Availability Statement

The original contributions presented in the study are included in the article/**Supplementary Material**. Further inquiries can be directed to the corresponding author.

## Ethics Statement

The studies involving human participants were reviewed and approved by Friedrich-Alexander-University of Erlangen-Nuremberg. The patients/participants provided their written informed consent to participate in this study.

## Author Contributions

LO’N: data analysis, manuscript construction. PH: data analysis. QL: data analysis. MI: data analysis. VS: data analysis. JR: data collection and clinical trial. AH: data collection and clinical trial. VA: manuscript, data analysis. IS: manuscript, data analysis. HE-G: manuscript, data analysis. GS: data collection and clinical trial. JW: oversight, manuscript, data analysis. All authors contributed to the article and approved the submitted version.

## Funding

The authors above have no relevant financial disclosures or benefits from commercial sources that could create a potential conflict of interest. The entirety of this work was funded by a grant obtained by JA Wilkins through the Canadian Foundation for Innovation.

## Conflict of Interest

The authors declare that the research was conducted in the absence of any commercial or financial relationships that could be construed as a potential conflict of interest.

## Publisher’s Note

All claims expressed in this article are solely those of the authors and do not necessarily represent those of their affiliated organizations, or those of the publisher, the editors and the reviewers. Any product that may be evaluated in this article, or claim that may be made by its manufacturer, is not guaranteed or endorsed by the publisher.
