# Supplementary material for: Proteomic Approaches to Defining Remission and the Risk of Relapse in Rheumatoid Arthritis
Source: Front Immunol. 2021 Nov 18;12:729681. doi: 10.3389/fimmu.2021.729681 (PMC8636686; doi:10.3389/fimmu.2021.729681)
Supplement: Supplementary file 1 [file DataSheet_1.docx]

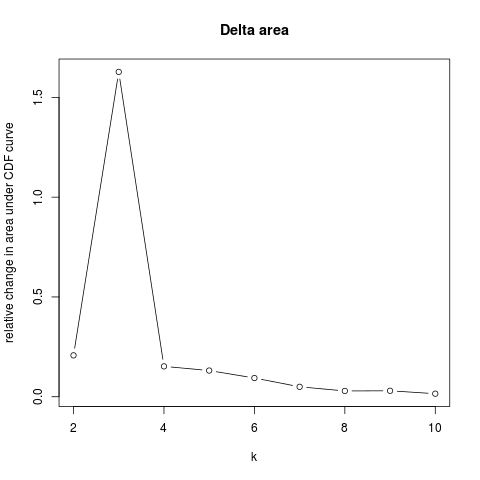


**Figure S1: Consensus cluster plus using 80% protein resampling and 80% patient resampling and selected Pearson as the distance function identified that greater than four clusters provided a relatively low change of cumulative distribution.**

**Figure S2: Multi-dimensional scaling plot of hierarchical clusters (left) and flare status (right).**

**
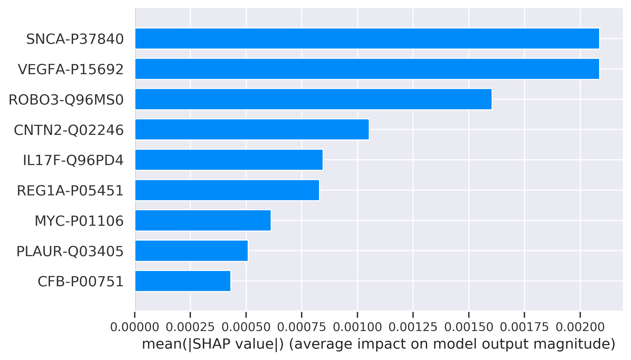
**

**Figure S3: Mean SHAP values which are indicative of importance and contribution to XGboost model to distinguish future flare versus remission.**


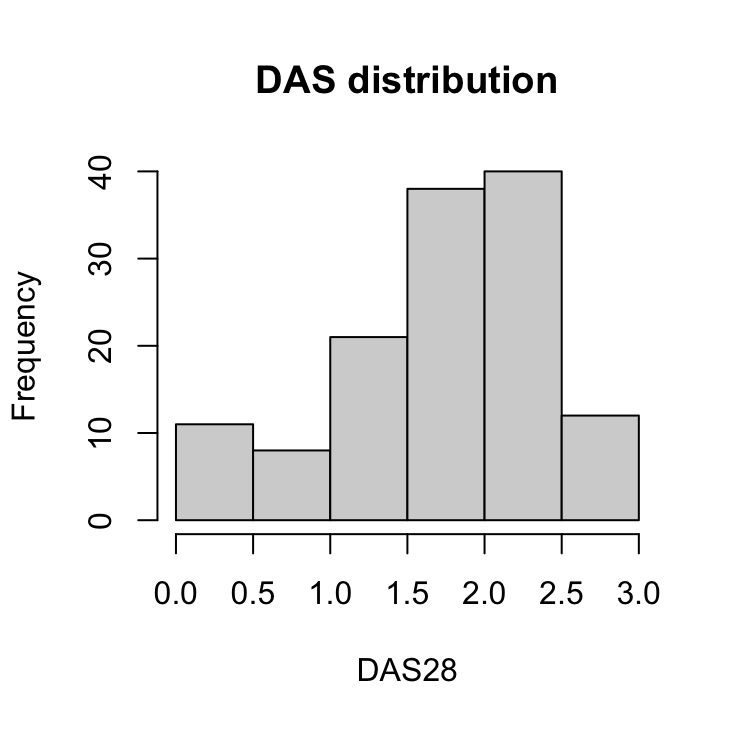


**Figure S4: Histogram of DAS28 scores amongst population of stable RA patients included in the RETRO study. DAS28: Disease activity score**

**
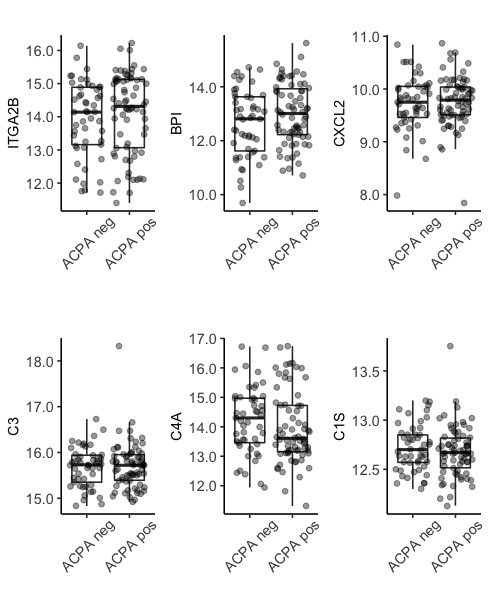
**

**Figure S5: No differences between ACPA positive and negative RA patients in multiple parameters included in DAS28 proteomic score.**


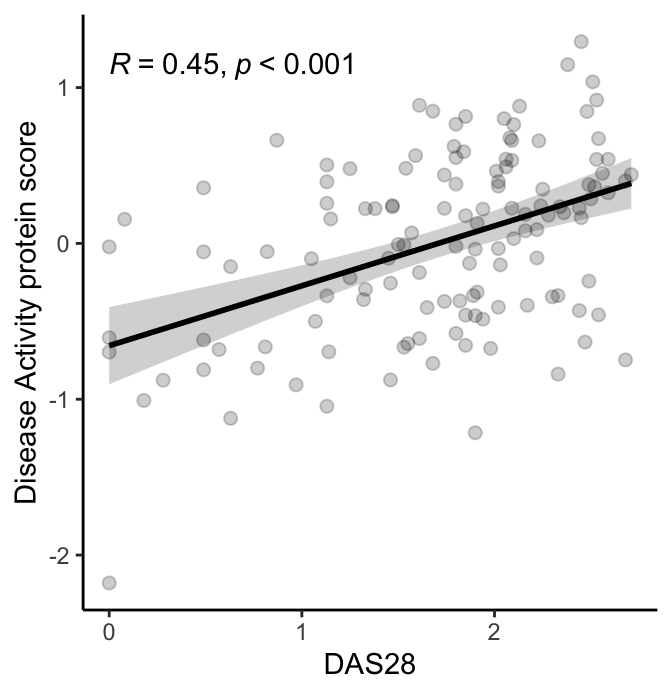


**Figure S6: Correlation between disease activity protein score and DA28 score. DAS28: Disease activity score**

**Figure S7: Random sampling of 5000 sets of 34 serum proteins and their correlation with DAS28 score, range is -0.37 to 0.27 (left). 4 examples of 34 protein sets and their correlation with DAS28 scores. DAS28: Disease activity score**

|  | Cluster 1 |  |  |  | Cluster 3 |  |  |  | Cluster 4 |  |  |  |
| --- | --- | --- | --- | --- | --- | --- | --- | --- | --- | --- | --- | --- |
|  | OR | 2.5 | 97.5 | p-value | OR | 2.5 | 97.5 | p-value | OR | 2.5 | 97.5 | p-value |
| HAQ | 24.84 | 0.15 | 4002.26 | 0.22 | 33.56 | 0.21 | 5241.26 | 0.17 | 19.11 | 0.11 | 3456.10 | 0.27 |
| DAS28 | 1.35 | 0.42 | 4.32 | 0.61 | 0.78 | 0.27 | 2.30 | 0.66 | 0.50 | 0.17 | 1.50 | 0.22 |
| Age | 0.96 | 0.91 | 1.02 | 0.22 | 0.97 | 0.92 | 1.03 | 0.29 | 1.00 | 0.94 | 1.06 | 0.95 |
| Sex | 0.82 | 0.17 | 3.81 | 0.80 | 0.54 | 0.12 | 2.37 | 0.41 | 0.38 | 0.08 | 1.73 | 0.21 |
| ACPA | 0.33 | 0.05 | 2.16 | 0.25 | 0.30 | 0.05 | 1.77 | 0.18 | 0.41 | 0.07 | 2.62 | 0.35 |
| RF | 5.62 | 0.88 | 35.97 | 0.07 | 2.58 | 0.44 | 15.18 | 0.29 | 1.97 | 0.31 | 12.38 | 0.47 |
| Disease Duration | 0.95 | 0.85 | 1.06 | 0.32 | 0.91 | 0.81 | 1.02 | 0.10 | 0.88 | 0.77 | 1.00 | 0.05 |
| Remission Duration | 1.01 | 0.96 | 1.06 | 0.69 | 1.01 | 0.97 | 1.06 | 0.56 | 0.99 | 0.94 | 1.04 | 0.66 |
| **Flare** | **3.66** | **0.61** | **22.19** | **0.16** | **5.67** | **0.97** | **33.06** | **0.05** | **4.85** | **0.79** | **29.75** | **0.09** |

**Table S1: Results from multinomial regression analysis and variables included in model. Cluster 2 is considered the baseline group. OR: Odds ratio.**

| name | Log FC | p-value | adj p-value |
| --- | --- | --- | --- |
| EDAR | 0.067 | 0.001 | 0.918 |
| EPHB2 | -0.022 | 0.004 | 0.918 |
| CX3CL1 | -0.022 | 0.004 | 0.918 |
| SPINT2 | 0.038 | 0.004 | 0.918 |
| NCR3 | -0.019 | 0.004 | 0.918 |
| AMICA1 | -0.025 | 0.004 | 0.918 |
| LGALS9 | -0.021 | 0.007 | 0.987 |
| GPNMB | -0.023 | 0.018 | 0.987 |
| CRTAM | 0.023 | 0.018 | 0.987 |
| CD80 | -0.015 | 0.018 | 0.987 |
| IFNA2 | -0.015 | 0.018 | 0.987 |
| ASAH2 | -0.028 | 0.019 | 0.987 |
| IGF1 | -0.022 | 0.021 | 0.987 |
| MIA | -0.019 | 0.022 | 0.987 |
| CD97 | -0.012 | 0.024 | 0.987 |
| NRG1 | -0.033 | 0.026 | 0.987 |
| DSCAM | -0.024 | 0.027 | 0.987 |
| SIGLEC14 | -0.069 | 0.027 | 0.987 |
| BCAN | -0.017 | 0.028 | 0.987 |
| TGFBR2 | -0.029 | 0.030 | 0.987 |
| IL5 | -0.040 | 0.031 | 0.987 |
| PAK3 | -0.017 | 0.037 | 0.987 |
| SIRT2 | 0.030 | 0.037 | 0.987 |
| CD47 | 0.021 | 0.038 | 0.987 |
| GNLY | -0.030 | 0.039 | 0.987 |
| IL4R | -0.026 | 0.040 | 0.987 |
| CTSA | 0.059 | 0.041 | 0.987 |
| SNCA | 0.063 | 0.045 | 0.987 |
| NRP1 | -0.014 | 0.045 | 0.987 |
| CSF3R | -0.017 | 0.047 | 0.987 |
| PTHLH | -0.026 | 0.048 | 0.987 |
| RPS3A | 0.023 | 0.049 | 0.987 |

**Table S2: Differentially expressed proteins in future Flare group compared to future remission group using *limma* linear model.**

| Feature | Importance |
| --- | --- |
| PEBP1-P30086 | 1.0345991 |
| HPX-P02790 | 1.03083622 |
| ANGPT4-Q9Y264 | 0.89181068 |
| EHMT2-Q96KQ7 | 0.68736361 |
| TFF2-Q03403 | 0.64036031 |
| ACVRL1-P37023 | 0.58347313 |
| EDAR-Q9UNE0 | 0.56621273 |
| SERPINE2-P07093 | 0.53127235 |
| GDF5-P43026 | 0.47068795 |
| SIRT2-Q8IXJ6 | 0.46754083 |
| IGFBP1-P08833 | 0.4213563 |
| HAMP-P81172 | 0.39173379 |
| TDGF1-P13385 | 0.37876234 |
| PSMA2-P25787 | 0.33174147 |
| CD274-Q9NZQ7 | 0.31378101 |
| CTSA-P10619 | 0.29889931 |
| EPB41-P11171 | 0.28053562 |
| FGF19-O95750 | 0.22041883 |
| CTSD-P07339 | 0.20636657 |
| GRB2-P62993 | 0.20073673 |
| CXCL8-P10145 | 0.14326146 |
| STIP1-P31948 | 0.11234916 |
| RPS3-P23396 | 0.10963818 |
| CHI3L1-P36222 | 0.10160687 |
| FCGR2A-P12318 | 0.08914953 |
| HBB-P68871 | 0.08288497 |
| HBA1-P69905 | 0.07883273 |
| NGF-P01138 | 0.07703274 |
| FAM3D-Q96BQ1 | 0.07224064 |
| FSHB-P01225 | 0.04541534 |
| TAGLN2-P37802 | 0.03117022 |
| CD47-Q08722 | 0.01468859 |
| IFNA10-P01566 | 0.00915226 |
| CRP-P02741 | 0.00859643 |

**Table S3: LASSO regression model with associated feature importance for predicting Flare versus remission.**
